# The development of mental health services within primary care in India: learning from oral history

**DOI:** 10.1186/1752-4458-8-30

**Published:** 2014-07-16

**Authors:** Nadja van Ginneken, Sanjeev Jain, Vikram Patel, Virginia Berridge

**Affiliations:** 1 Centre for Global Mental Health, London School of Hygiene and Tropical Medicine, and Sangath, LSHTM, Keppel Street, London WC1E7HT, UK; 2 Sangath, Porvorim, Goa, India; 3 National Institute for Mental Health and NeuroSciences, Bangalore, India; 4 Centre for History in Public Health, London School of Hygiene and Tropical Medicine, London, UK

**Keywords:** Mental health, History, India, Developing countries, Health policy, Health planning, Primary health care, Health workers

## Abstract

**Background:**

In India very few of those who need mental health care receive it, despite efforts of the 1982 National Mental Health Programme and its district-level component the District Mental Health Programme (DMHP) to improve mental health care coverage.

**Aims:**

To explore and unpack the political, cultural and other historical reasons for the DMHP’s failures and successes since 1947 (post-independence era), which may highlight issues for today’s current primary mental health care policy and programme.

**Methods:**

Oral history interviews and documentary sourcing were conducted in 2010–11 with policy makers, programme managers and observers who had been active in the creation of the NMHP and DMHP.

**Results:**

The results suggest that the widely held perception that the DMHP has failed is not entirely justified, insofar that major hurdles to the implementation of the plan have impacted on mental health coverage in primary care, rather than faults with the plan itself. These hurdles have been political neglect, inadequate leadership at central, state and district levels, inaccessible funding and improperly implemented delivery of services (including poor training, motivation and retention of staff) at district and community levels.

**Conclusion:**

At this important juncture as the 12th Five Year Plan is in preparation, this historical paper suggests that though the model may be improved, the most important changes would be to encourage central and state governments to implement better technical support, access to funds and to rethink the programme leadership at national, state and district levels.

## Background

In low- and middle- income countries (LMICs) very few mentally ill people receive mental health care despite available evidence for cost-effective and feasible packages of care [1,2]. The scarcity of specialist human resources, as well as large inequities and inefficiencies in resource allocation are significant reasons why this treatment gap remains [3,4]. Currently available studies from LMICs suggest various primary health care worker (PHWs) cadres (primary level doctors, nurses, lay health workers and other generalist paraprofessionals with no specialisation in mental health) are effective in a range of interventions for mental, neurological and substance abuse disorders [5]. In light of achieving universal health coverage, efforts at a global level and within India have advocated task-sharing and better leadership in scaling-up services [6]. In particular, the WHO Mental Health Gap Action Programme created guidelines for task-sharing mental health interventions with non-specialists [2,4,7].

India was the first post-colonial “non-white” independent country to have mental health reforms. The national mental health programme (NMHP), created three decades ago in 1982, established an integrated approach to mental healthcare delivery utilising a specialist and non-specialist workforce. There is a widely held perception that the NMHP failed [8]. Mental healthcare coverage has certainly been limited on both the specialist and the primary care fronts. There are 3600 psychiatrists in India for a population of 1.2 billion [9]. Most are located in the private sector and in major cities. There is a 40–60 fold deficit in the number of clinical psychologists, social workers, and nurses [9]. As for primary mental health care, still only 127 districts of the 626 districts in India have implemented the District Mental Health Programme (DMHP), the district implementation of the NMHP which operationalises mental healthcare integration into primary care. Within these districts not all primary care doctors are trained [10].

The aim of this study is to explore and unpack the political, cultural and other historical reasons for the DMHP’s failures and successes since 1947 (post-independence era). At this important juncture, as a 12th Five Year Plan is in preparation, which is the sixth Five Year Plan since the NMHP started, this historical analysis is critical to policy makers when rethinking the current DMHP’s implementation.

## Methods

The first author (NvG) conducted oral history interviews in 2010–11. This marked the end of a government health planning cycle, the 11th Five Year Plan. Oral histories are in-depth interviews with witnesses to and participants in past events. The method captures individual memories and thus personal and social perspectives on events, which can be crucial in complementing written documentation. It may be the only recording of certain events which have no written evidence. This study interviewed the ‘elite’ (such as civil servants and professionals) to better understand policy and political processes, and the interplay with personalities [11].

To select interviewees, five contacts known to one of the authors (VP) helped identify further participants through ‘snowballing’. Of 26 potential interviewees, 17 were purposively selected to represent different perspectives, backgrounds and time periods. They comprised national and regional Indian mental health policy makers, clinical experts and programme implementers, sometimes fitting into multiple categories (see Table 1), who were active between 1975 (when WHO advocated the extension of mental health services) and the present day. These audio-recorded interviews were conducted in English, and followed a narrative of each individual’s involvement in mental health policy and programmes in India, what they viewed as current key issues and the future vision for improving mental healthcare in primary care. Informed consent was obtained from all participants.

**Table 1:** Participants characteristics (n = 17)

**Roles**	Numbers*	**Details**
Clinical psychiatrists	14	• Six retired
		• Eight implemented mental health programmes
		• Nine advisors/decision makers (state or central government)
		• Three work within NGOs
		• One private psychiatrist
		• Four now work abroad
Bureaucrats	7	• Five bureaucrats within the Indian Government
		• Two international-level bureaucrats
Programme implementers	9	• Six NGO programme founders or coordinators, of whom one user-survivor
		• Four government programme implementers
Academics	8	• All did research in India
		• One lawyer, seven psychiatrists

*most participants had two or three different roles so numbers do not add up.

Written historical material was gathered from literature searches and participants. We relied principally on oral history sources and published materials (Indian newspapers, training manuals, government reports) as has been done in other contemporary history studies [12]. Indian organisations such as mental health institutions, psychiatric societies, or NGOs who were approached have not maintained formal archives. Correspondence and records of formal governmental reviews are not available in the public domain. Many documents had been destroyed from lack of space, or other administrative reasons. Some documents, the author was informed, were retained in various professors’ offices. Through attempts to track these through participants and their contacts, the first author obtained access to some unpublished material such as minutes of meetings, grant reports, unpublished papers, memorabilia related to the organisation’s activities, but not to any administrative records.

The main analysis focussed on the interviews which were transcribed and coded. The codes were analysed within a thematic framework which combined deductive themes (present in the interview guide), as well as inductive themes (identified during the process of coding). Written sources helped to cross check and contextualise emerging data to highlight discrepancies and inconsistencies in interviewees’ memories of events and processes. This methodological triangulation allowed the identification of critical perspectives and emerging themes [13], and identified the important time periods of mental health policy developments.

Themes inductively identified in the analysis matched the existing functional typology of health system policies [14,15]:

1. Delivery arrangements (which services, to whom, by whom, what settings and accessibility, health information and technology, supplies, quality and safety monitoring mechanisms)

2. Financial arrangements (financing of the programme, funding of clinics for services, remuneration of providers)

3. Governance arrangements (establishments of responsibilities and accountabilities at the levels of policy and professional authorities and consumer/stakeholder involvement in policy decisions)

Ethical approval was gained from the London School of Hygiene and Tropical Medicine, from Sangath, Goa, and from the Indian Medical Research Council. Consent was obtained from all participants.

## Results

An overview of the recent milestones of primary mental health care developments in India is presented to set the context for the second section of the results which will explore the reasons for achievements and failures of the DMHP.

### A brief overview of phases

The overview starts from Independence of India (1947) to set the full context of primary mental health care developments. This study identified seven key periods (Table 2), which were similar to other NMHP historical reviews [16,17]. These time periods delineated the rise of the NMHP, its fall in the 1990s, and a recent rise of government attention to the NMHP in the 21st century.

**Table 2:** History of mental health care integration within the Indian health system*

**Time periods**	**Date**	**Health system and political developments**	**Mental health developments**
PRE-INDEPENDENCE	early 20th century	1935 Act: provinces autonomy for Health activities	Growth of mental hospitals, first general hospital psychiatric unit (GHPU)
	1946	Bhore Committee Report	
1. POST- INDEPENDENCE	Aug 1947	Independence of India declared	
	1950s	1st Five Year Plan (FYP)	1950s: Psychotropic medications developed
			1954: All India Institute of Mental Health (AIIMH) established, Bangalore
	1956	Second FYP. Rs. 225 crore (5%) for health	Late 1950s: concept of ‘family ward’ (Amritsar and CMC); nurse training at AIIMH
	1961	3rd FYP. Rs. 342 (4.3%) for health; Mudaliar Committee Report	1960s: More GHPUs and specialists; psychiatric social worker training in AIIMH
	1969	4th FYP. Rs. 840 crores for health	
	1973	Medical personnel forced to work in rural areas; Multi Purpose Workers introduced; 1974: 5th FYP. Rs. 796 crores health	1974:NIMHANS replaces AIIMH and the government mental hospital
2. PILOTING MODELS FOR MH CARE EXTENSION	1975		WHO report on organisation of mental health services; Community Psychiatry Unit created in NIMHANS
	1977	Community health workers and Dais	1975-1981: WHO: ”strategies for extending mental health care” (including Raipur Rani)
	1978	Declaration of Alma-Ata	1975-1986: Sakalwara – NIMHANS model. Other similar projects: Delhi, Jaipur, Hyderabad
	1980	6th FYP	
3. NMHP- INITIAL STEPS	1982	National Health Policy	National Mental Health Programme initiated. Budget: 10 million rupees for the first 5 years
	1985	7th FYP	Bellary programme (1985–1990)
	1987		Mental Health Act
4. POLITICS, POWER and NGOS	1990s		Increasing number of NGOs. E.g.: 1993: Banyan; 1996: Ashadeep, Sangath, GASS; 1999: Bapu Trust
	1992	8th FYP	Community mental health featured on health budget
	1994		Persons with Disability Act
5. DMHP/HUMAN RIGHTS	1996		DMHP implemented. Budget: 270 million rupees;
	1997	9th FYP	
	1998		The National Human Rights Commission Report
	2001		Erwadi disaster (Tamil Nadu)
6. RESTRATEGISED NMHP	2002	10th FYP; National Health Policy	Re-strategised NMHP. Budget: 1.9 billion rupees
	2004	National Rural Health Mission. ASHA worker created.	
	2005		UN Convention on the Rights of People with Disabilities
7. REINVIGORATED DMHP	2007-2011	11th FYP	2007: ‘Reinvigorated’ NMHP. Budget: 10 billion rupees

*based on findings of interviews and references: [16-20].

1. *1946 to 1975: Creating an Indian system of mental health care*

The evolution from asylums to more humanistic mental health institutions began in the 1920s. Significant developments – internationally (psychotropic medicines) and in India (General Hospital Psychiatric Units, more specialists and epidemiological surveys) - contributed to mainstreaming psychiatry as a medical speciality. The post-Independence government focussed mainly on psychiatric training and building hospitals rather than on developing a non-mental health specialist workforce as intended by the Bhore Committee Report, a report set up by the colonial government, headed by Sir Joseph Bhore and advised by a panel of international experts, intended to address the health needs of India in a post-colonial era [21,22]. In the 1950s and 1960s non-mental health specialists were used only in a handful of tertiary care settings (Amritsar, Madras and Calcutta). No formal government plans existed for extending mental health services to the community. However this was a major time for the development of primary care and community health worker services in general [23].

2. *1975 to 1982: piloting models for extending mental health services*

The WHO’s study, “*Strategies for extending mental health care*” [24], instituted primary-level health worker (PHW)-delivered mental health care in seven countries. One site was in Raipur Rani, northern India (1975–81). A similar model was developed in Karnataka, southern India (1976–1986) through the National Institute for Mental Health and Neurosciences (NIMHANS), one of the largest mental institutions in India, and one of the few heavily involved in national mental health planning and implementation. Twenty nine other minor similar models emerged across the country [25].

Inspired by these apparently successful models and by primary care developments (1978 Alma Ata Declaration, primary care in India), a small taskforce committee produced a National Mental Health Programme (NMHP), which was adopted by the Government of India in 1982. The NMHP was initiated to promote community mental healthcare through an intersectoral approach and through integration with primary care by training existing PHWs to diagnose and treat mental disorders. The NMHP programme, though conceived as one plan, evolved in nature and remit according to decisions taken at the beginning of each ensuing Five Year Plan.

3. *1982 to 1990: the NMHP’s first steps*

In the early 1980s, NIMHANS identified that their models which operated at PHC level were too resource-intensive for a small catchment area. They therefore piloted a district-level initiative in the Bellary district in Karnataka State (1985–1990) [26]. Simultaneously, the NMHP asked each state to *“operationalise a programme in at least one district in their State*” [25]. The Bellary model, one of the few operationalised and favourable programmes, was taken up by the government as a national model and has remained the model for primary mental care delivery ever since.

4. *1990 to 1996: Politics, power and the rise of NGOs*

The NMHP continued to be hospital-focussed [27]. During these years, the healthcare system in India moved away from the 1982 pro-poor and comprehensive National Health Policy and this development also coincided with a faltering of the comprehensive ideology of Alma Ata. The government reduced the healthcare budgets of the States [28] and this affected mental healthcare. Earlier community mental health models (e.g. Raipur Rani) collapsed and their leaders moved abroad. Few regional centres other than NIMHANS implemented the NMHP, and the programme stagnated.

NGOs thus flourished in order to address the gap in mental healthcare provision [29]. These developed several innovative models, including rehabilitation and advocacy, using an array of non-specialist health workers (such as social workers and users) and bypassing government primary care centres.

5. *1996 to 2002: The human rights agenda and DMHP creation*

Human rights violations in psychiatric and religious institutions were exposed through the media (27 chained mentally ill burned to death in an accidental fire in the Erwadi Dargah in 2001), by the Supreme Court (an evaluation of mental hospitals’ poor standards [30], and by human rights lawyers and activists. The human rights movement vilified institutional care. This helped the District Mental Health Programme (DMHP), launched in 1996, to gain support. The DMHP strongly advocated community care as part of the comprehensive integration of tertiary, secondary and primary care.

6. *2002 to 2007: The 10th Five Year Plan*

The NMHP in the 9th Five Year Plan had only focussed on the DMHP, so the 10th plan ‘restrategised’ the NMHP to strengthen and modernise state-level administration, mental institutions and medical colleges [31]. Few changes were made to the DMHP. New government officials were however favourable to the NMHP and increased its budget seven-fold, even though these funds were subsequently under-spent. A large private mental health sector flourished because of continuing poor government provision.

7. *2007 to 2011: the 11th Five Year Plan*

The NMHP was ‘reinvigorated’, following some adverse evaluations of the NMHP/DMHP [31,32]. With a budget increase to 10 billion rupees (still only 2% of the public health expenditure in 2007), new elements were incorporated into the NMHP such as school and suicide prevention programmes. Training of general medical officers became a priority.

### What have been the reasons for the achievements and failures of the DMHP?

The oral history interviews and information from documentary sources highlighted both ongoing and enduring issues which have affected the implementation of primary mental health care. Three key areas were identified: governance, financial and delivery arrangements.

### 1. Governance arrangements and leadership

Since the start of the NMHP, leadership and government commitment have been poor, and have lacked transparent and accountable systems. The reasons for this are presented below.

#### ***Inadequate leadership***

Firstly, respondents generally agreed that the government had neglected mental health and failed to adequately integrate it into their agenda.

*“It was never regarded as sufficiently important.[….] I don’t believe it was a conscious decision that ‘no, we do not need a mental health policy’ – it is just indifference”* (bureaucrat 1).

The apathy of central and state governments meant that the NMHP was dormant, “*mainly remaining on paper till the 1990s*” (psychiatrist/former leader 2). Governments never saw mental health as a public health problem. They were not proactive in mental health planning, certainly not when compared to other health sector planning, such as the family planning programme which started with strong leadership and had a policy in place by 1976 [33]. Despite several meetings with the Committee of the Ministry of Health and Family Welfare particularly throughout the 10th and 11th Five Year Plans, there was little progress in achieving their recommendations. For example from 2000 through to 2010 grant reports mention the problem of getting State level cooperation, but no action was ever taken. Only in 2010 did the report mention that “*the Department needs to take a proactive approach to bringing States onboard*” [34]. Even this remained a very vague statement rather than a solution.

The interviews concluded that that national leadership of the NMHP had been absent since the start of the programme. Establishing a central leadership was never a government priority because of the federal system - health is run as a central programme, but implemented by the states. This system of devolution derived from the colonial system of “not interfering with local initiative*”* was often seen as a subterfuge resulting in poor national and state level coordination and integration [35]. The NMHP initiators modelled the programme on the Bhore report and WHO technical recommendations but largely ignored the recommendations to create stronger central leadership as they focussed on local implementation.

Central leadership had been most obvious in the early years of the NMHP. The early community project leaders (1975–82), and the next generation at district level (1982–90) by default also constituted the national leadership. These leaders recognised that they were overburdened by their multiple responsibilities and were therefore unable to commit the time to strengthening the NMHP.

*“So, I could not spend so much time. But since I had interest in [community mental health], I spend extra time, travel, then we developed a district program, and so on. That was all in addition to whatever we were expected to do as faculty, which is being an examiner, take lectures, and grand rounds and teach students.”* (psychiatrist/former leader 1)

In the 1990s, these NMHP leaders withdrew from the programme to pursue jobs abroad which they explained was not because they lacked commitment.

*“Some of us who felt passionately about this were marginalised for various reasons. This happens in India very often that if you are not in the favour of the authorities, your technical capacity does not have any meaning, it is only if you are occupying a particular position*” (psychiatrist/former leader 2).

The Indian hierarchical political environment which included more clinically and biomedically-oriented leaders at NIMHANS and within government, wore down the “*perseverance and political persuasion*” (psychiatrist/international leader 5) of community psychiatric leaders. Published papers and NGO reports confirm that the petty politicking and patronage amongst public health leaders has been a widespread feature in India, with very few examples of Basaglia, Beveridge or Freire social or inclusive pro-poor ideology [36]. Those who did practice pro-poor ideologies also felt that their “unwanted human rights voices were silenced” [37].

A government advisor from the 1990s also recognised that leadership lacked continuity and was perhaps misguided. He felt they were “*on the wrong track*” (psychiatrist 7) and repeated mistakes from the 1980s. He “*re-learned*” that the DMHP model’s top-down approach inadequately addressed the ground realities of attrition, poor supervision and utilisation of PHC services [8].

Previous leaders expressed the view that in the last 10 years central leadership had declined because of a lack of sufficiently motivated psychiatrists, and because others had been attracted to the private sector. Government reports also stated that the “dismal performance” of the programme was for these reasons [34].

State-level and local leadership had always been poor. In the 1980s various psychiatrists ran workshops to try to encourage state and district administrators and finance officers to implement the NMHP [38]. These efforts failed to kick-start local leadership and were discontinued in the 1990s. Most respondents suggested that training alone was insufficient.

*“So, it is not a lack of technology or know-how of reducing or preventing the illness – it is the delivery. Everything depends on the leader; there is a lack of leadership in many places – the District Health Officers are not convinced that this is one of the priority programmes. […] At the State Annual Review, there has to be a review of Mental Health; it has to percolate down. If you just train somebody and leave it at that, it is not going to help. That has not occurred.”* (psychiatrist/former leader 1)

The NMHP model was not adapted by states’ departments of health because they were expected to adapt and initiate the programme without receiving adequate incentives or technical support.

Furthermore, a prior national bureaucrat/ psychiatrist felt that because earlier NMHP projects’ leaders (from Raipur Rani and Bellary) used top-down and oligarchic leadership methods, this led to these projects’ demise.

*“Those were not dynamic people, they did not have energy […] they did not involve people.[…] When it is an individual centre, it does not survive - when it is a community centre, it survives […]. Many people would like to be too egoistic to develop that model.[…] We must learn how [to] change their models to suit the needs of the community.” (*psychiatrist 4)

One respondent suggested that these projects were unsustainable because the authoritarian approach of local programme leaders harmed the reputation of the community programmes.

*“Influential people in rural communities were given better care at home and in hospital by senior leaders, while the poorer were seen by juniors.”* (psychiatrist 14)

These personality-driven approaches and these examples of favouritism within the community were antithetical to the values of community care, where one may expect an egalitarian service to reduce rather than reinforce inequalities in provision. This non-democratic process caused much cynicism amongst psychiatric and medical professionals.

#### ***Accountability and transparency***

Certain system weaknesses were identified through internal evaluations [39,40] but were largely ignored. Respondents acknowledged that no mechanisms existed to make authorities accountable for addressing identified weaknesses.

*“The biggest problem was that we did not develop indicators. That is the limitation of all health programmes in India except TB:[…] they look at it and see […] if corrective action is possible. In the District Mental Health Programme, no corrective action has been taken”* (psychiatrist/former leader 1)

For example, as mentioned in interviews and in the literature, no evaluations assessed patient recovery indicators (psychiatrist 13) [41]. A former government adviser explained the lack of central government ability to intervene:

*“Because, health is a state subject we can’t interfere with the health aspect of any State.[…] We can provide the money, we can provide the guidelines but, we can’t call them to task, we can’t hold them accountable.”* (psychiatrist/former bureaucrat 7)

Government reports also highlighted the system lacked mechanisms to penalise health workers’ non-performance (in any area of healthcare), or to make them legally accountable [10]. This contributed to poor service provision.

#### ***WHO’s influence in setting up the NMHP***

In the early years the most important influence was the WHO’s mental health department.

*“Health is the weakest element of the Government of India.[…] [The WHO] was trying to make [the various ministers] do things, use the authority of WHO to promote the programmes that have been composed and that have been accepted by the Government”* (psychiatrist/international leader 6).

Indeed, India as well as many countries, were influenced by the WHO. Though some critiques have suggested WHO’s hegemony is a form of neo-colonisation, circumstances here were different. Since the 1960s, Indian psychiatrists worked within the WHO mental health department and influenced their strategies. Indian leaders at the time thought WHO’s input was essential.

*“But for WHO support, local ministry of health would have never made the National Programme of Mental Health. This was because WHO has supported it, they were willing to look at it.”* (psychiatrist/former leader 3)

#### ***Participatory and inclusive decision making***

The stagnation of the NMHP in the 1990s was associated with a dearth of external lobbying groups. However in the late 1990s and early 2000s several human rights outcries pushed the government into a judicial intervention [42]. The most influential outcries were created following the release of the 1999 National Human Rights Commission which addressed poor standards of care in mental hospitals, and much more importantly following the media outcry over the Erwadi tragedy.

Grassroot leaders such as established NGO leaders, tried to partake in government-level decision making. They felt their efforts were unsuccessful.

*“[Our NGO] is not working with the DMHP […]. We are trying to link up with them, but that’s entirely different thing.”* (psychiatrist/former NGO leader 11)

In return, bureaucrats were met with often radical and conflicting suggestions, which ignored contemporaneous government priorities, from fragmented mental care stakeholders.

*“Policy planners sense dissonance in the group and this gives them a reason not to take action”* (psychiatrist/NGO leader 12)

A current government official recognised the government’s inaction to date, but also recognised NGOs’ untapped potential.

*“Government has not yet got around to recognising [NGOs] as training centres. I believe […] that we have to recognise that these are institutions that have been able to establish a model of community-level care”.* (bureaucrat 1)

Recently, more effort to involve different lobbies, such as in the recent revision of the Mental Health Care Act, has occurred. The challenges highlighted in the 10th plan mentioned for the first time the need to “harness NGOs’ help in community based care of mentally ill” [43]. Engagement of consumers within the public sector however is still non-existent.

### 2. Financial arrangements

#### ***Funding in the NMHP’s early years***

The 1970s pilot projects were well funded (10 million rupees) by NIMHANS and the WHO, as were the early years of the NMHP.

*“NIMHANS was totally committed in the ‘70s and ‘80s [so] the programme went so quickly. When there was no money- the District Mental Health Programme came up without the NMHP money – it came up with the local money like the Government of Karnataka, [and] the NIMHANS local funds.”*(psychiatrist 2)

With an increasing unfavourable international financial climate in the late 1980s, the WHO withdrew their support for their pilot project. Changing priorities within NIMHANS meant their pilot programme funding also dwindled. However central government budgets increased:

*“Now, people were beginning to realise that unless you invest in basic health care in rural areas, things are not going to change.[…] So, for the first time in 1996, the Government of India health budget, community mental health figured. They accepted it for the district mental health programme delivery. And subsequently, money has never been a problem.”* (psychiatrist 1)

#### ***Financing hurdles in the last decade***

Since the 10th Five Year Plan (2002) the budget has been more realistic (1.9 billion rupees in the 10th plan, and 10 billion rupees in the 11th). These amounts unfortunately have been under-spent because of *“jurassic financial procedures”* (psychiatrist 7), a common occurrence in the health sector.

*“Money is there but it cannot be used, as the person who has to sanction it sits in Delhi.”* (psychiatrist 1)

Committee reports throughout the 9th, 10th and 11th plans have mentioned that central fund allocation was often consistently reduced by at least half of the estimated amount because of under-spending, and actual expenditure was often even less. For example in 2002–2003, the first year of the 10th plan, the initial plan was to spend 300 million rupees. Due to previous under-spending only 35 million (one tenth of planned spending) was finally allocated. Of this only 900000 rupees (2.5% of allocated spending) was spent [44,45]. The early reports tended to blame State governments for under-spending because they “failed to forward their proposals without delays”[45], and “the Department [was] in non-receipt of complete proposals from State governments and institutions” [43]. However the Committee reports also recognised and confirmed what policy makers stated, that administrative bottlenecks occurred at central government level which also contributed to inability to access funds. Expenditure on new DMHP plans (such as extending the plan to new districts) was frozen for the first two years of the 10th and 11th plans as the central government had not approved these proposed changes [45,46]. Also decisions on yearly spending were often delayed by holding funding meetings shortly before the end of the financial year [34,44]. These barriers have never been overcome, and continue to appear in more recent reports on the NMHP [10,34]. No solutions have been suggested apart from one vague statement that the “department needs to take proactive approach to bring States on board” [34]. These financing issues are to be found across the health sector, not just in mental health [34].

Fund allocation within States has also been poor. Less than 1% of the total health budget was allocated to the NMHP in the North-Eastern States of India [44]. Across all States, DMHP staff’s low and often delayed remuneration has compounded the problem of attracting and retaining specialists.

Financing has also been subject to petty politics. A former bureaucrat mentioned how power games blocked certain applicants:

“*Here were unexpected hurdles,[…] we had excellent research proposals, but again, due to obstructionist tactics,[…] most of the research proposals […] were blocked”* (psychiatrist 7).

Because of the consequent under-spending of the budget, the NMHP lost credibility with the Planning Commission. Funds were disbursed to other programmes like the National Rural Health Mission (NRHM) in 2005. The 11th plan’s funding was submitted to increased bureaucratic hurdles to regularly review performance and spending.

### 3. Delivery arrangements

Interviewees debated whether the DMHP model was appropriate in terms of its organisation of services and human resources.

#### ***Organisation of services at PHC level***

Certainly in the early years, the NMHP was described by participants and the literature as advanced in its thinking because it was one of the first LMIC mental health programmes. NIMHANS was responsive and proactive when scaling up from primary care to district level was required. It also had positive outcomes for patient detection and symptom reduction [26].

#### ***Criticisms of the Bellary (DMHP) model***

The Bellary model was intended to extend coverage in the northern part of Karnataka State, and had heavy psychiatric input (psychiatric outreach camps) from NIMHANS. As a Bellary programme founder explained, this model was utilised after its initial evaluation for a different aim, as a DMHP pilot for national coverage:

“*It was very important to recognise that the goal was not that we would be able to reach everyone - universalised coverage; it was increasing coverage – say from 5-10% or nil, to as much as possible. This is a very important thing that needs to be recognised because if we are thinking of universal coverage, then what we were achieving was totally inappropriate.*” (psychiatrist/former leader 2)

Because the motivated new NMHP taskforce were keen to start a model, they pushed forward one of the few models in existence in India.

A Bellary programme founder questioned however why, if the model was not designed with national coverage in mind, the NMHP had continued “*picking up the skeleton*” of the same model (psychiatrist 2). The only adaptation was to reduce psychiatric support and PHC doctors’ length of training which proved to be detrimental. There was very little questioning of whether overburdened, poorly utilised PHCs within weak health systems [47] should continue to be the DMHP’s main delivery mechanism.

This model was further criticised for its sole focus on medication. Jain and Jadhav [48] argued that the pill provided a ‘technical fix’ that policy makers required to fund and popularise the programme, whilst psychosocial interventions were ignored. A human rights lawyer felt the overmedicalised model was harmful.

“*The National Mental Health Programme has very limited imagination. It did not escape the medical paradigm. Whereas mental health needs […] has a much larger range: […] social injustice, […] torturous conditions at work, less than minimum wages, […] precipitators of poor mental health. Instead of addressing those structural questions we believe that we’re going to give people psychotropic medication and going to set things right. It’s hugely dangerous in a poor country*.” (lawyer 1)

A senior advisor of the 10th plan defended these decisions as successful cost reduction of psychotropic drugs had made these affordable and cost effective solutions for the government:

*“If I had got involved in the other thing [psychosocial interventions], we could not have got involved anywhere; because the bureaucrats want cut and dried, black and white things, you see. They can’t appreciate shades of grey.”* (psychiatrist/former bureaucrat 7)

Though the overmedicalisation critique is valid in essence, there were reasons for the ‘technical fix’. Policy makers were not ready to accept wider changes and innovations. In addition, funds were limited and thus minimising costs was important. Furthermore there was a growing international evidence base for antidepressants and antipsychotics (randomised controlled trials, systematic reviews) and treatment algorithms, and very limited evidence for non-pharmacological interventions [49].

Hardly any cultural or religious paradigms filtered down to community mental health care [41] and some respondents felt that, hospital and community psychiatric care had remained insufficiently ‘Indianised’. The creation of the NMHP was preceded by several decades of controversy over the western versus indigenous medicine debate. At the time the Bellary model was created, few allopathic doctors’ supported integrated approaches with other medical traditions, as a recent attempt to train ‘integrated doctors’ in both medical paradigms had failed [50].

#### ***Poorly motivated and trained health workforce***

Throughout the NMHP’s three decades, building a rural mental health workforce only involved PHC doctors training. Very little was initiated to help psychiatrists adapt to their new supervisory roles.

a. Primary care doctors

Early pilot project leaders explained the initial challenge in the 1980s was to train a new human resource, the PHC doctors.

*“This was a great challenge, […] so, how to train the health worker, what are his responsibilities, can we do it, how to monitor them, what kind of supervision do they require, […]. Whether it succeeded or not is a different story, and that is the next 20 years’ story.”* (psychiatrist 1)

As suggested by this psychiatrist, their initial package was comprehensive but as the model was scaled up in subsequent years, the reality of health workers’ context and qualities soon disrupted this plan. One contributing factor was PHC doctors’ large workload.

“*I met primary health care doctors and universally they said, that in the existing state, it was an additional burden – it was not doable, although they were trying their best to do it. So, I could make out that the original concept of Bellary was no longer suited*.” (psychiatrist/former bureaucrat 7)

Retaining doctors in rural areas and their frequent transfers was also a problem [51]. Furthermore, a bureaucrat explained that PHC doctors’ competency reduced since independence, making them more difficult to train, motivate and retain.

*“The increase in the number of medical colleges and private medical colleges has meant that the quality of teaching has suffered. […] The result of this is that a very indifferent quality of doctor is coming out of the medical education system. The best amongst these are probably staying in the cities. […] The GP [family physician] in India pre-independence, […] came through a much better education system.”* (bureaucrat 1)

Despite some international evidence that primary health workers could effectively diagnose and treat mental illnesses [1], in India and elsewhere, PHC doctors only recognised between 20 and 40% of all mental illnesses [40,52]. The DMHP- and other health sector- planners ignored recommendations to evaluate primary health workers’ impact on patient outcomes [49].

Respondents suggested PHC doctors were never properly trained.

*“Training has been a token gesture for the departments of health to be ‘seen to be doing’ something.”* (psychiatrist 14)

The training manuals produced in Bangalore and Delhi were too complex and not properly adapted. The NIMHANS PHC doctors manual, rather than being clearly focussed on the main issues in primary care, synthesised psychiatric and psychology textbooks. They became more complex throughout the editions from 1985 to 2009 [53-55]. For primary care officers with no or little previous exposure to psychiatry, these increasing details were overwhelming and could not be integrated into their current practice. The same was true of the Delhi manuals [56]. Furthermore the manuals produced for community health care workers focussed on diagnoses and health worker behaviour, but had no useful information on how to support the family or patient, or the process of referral [57,58]. These manuals were written by specialists at NIMHANS, who did so without evaluation of previous training or consultation with the primary-level health workers.

In addition, the delivery of training was never adequate, and ongoing training reduced over time. In the early years, though initial training was short, there was informal and organised follow-up of PHC doctors by psychiatrists during their outreach activities. In the last decade, only the training component remained, and this continued to be short and didactic (only 15 days in Karnataka for example) or non-existent (in the northern States).

More important than the content of training was the lack of ongoing support to PHC doctors – again a chronically neglected problem.

*“As long as continuous support and supervision is not there, they will not perform, or you will not get the outcome.”* (psychiatrist 1)

A prior leader suggested this support was absent because of supervisors’ indifference to mental health which lead to demotivated primary care staff.

“*If the health authorities higher up […] do not take [mental health] seriously, they consider it’s useless and all that, then the lower staff also loses interest. […] Most of them have been untrained and they consider it just a fashion.”* (psychiatrist/prior leader 3)

b. Specialists

Since the NMHP’s beginning, there were too few specialists interested in supervisory work. This problem remained unchanged. From 1981, NIMHANS ran several ‘Training for Trainers’ workshops to train specialists in their new supervisory roles but by 1986 only 63 Indian psychiatrists were trained. By the 1990s this training programme had stopped [38]. Motivating psychiatrists to remain in community programmes was also a challenge. For example, those involved in the NIMHANS primary care pilot project requested to return to NIMHANS jobs after two years’ work in the programme (psychiatrist 14).

Specialists’ lack of involvement could have been due to their poor remuneration. Many psychiatrists also lost faith in this model because they felt PHC doctors’ limited training would be insufficient to provide adequate care. Psychiatrists have been reluctant to associate with other mental health professionals under the same umbrella term of ‘specialists’ probably because of a strong hierarchical structure within hospital care. A psychiatrist involved in the Mental Health Care Act revision observed:

“*We have created a category called mental health professional [which] includes a psychiatrist, a psychologist, a psychiatric nurse and a psychiatric social worker.[…] Now the psychiatrists are extremely angry about it because they see themselves now being equated with the other professionals.*” (psychiatrist 10)

For example psychiatrists quashed recent attempts by psychologists to lobby for greater prescribing powers and representation in decision-making. Such current tensions between mental health professional groups suggest more groundwork and involving them in decisions may be required before they accept shared responsibilities, for example in supervising primary care workers.

## Discussion

These oral histories and documentary sources have given insight into the achievements, limitations and personal struggles involved since the 1980s in trying to increase mental health coverage in India. The national programme’s basic model of delivering community mental health care through district hospitals and PHCs, a model commonly seen in high- and low-income countries, certainly followed the WHO 1975 recommendations of extending mental health services. It also has had similar aims to the currently favoured universal health coverage approach: to improve the quality, funding and equity of care [6]. In an attempt to answer our main question of why the DMHP has not succeeded in achieving its aims, several reasons have emerged. The NMHP was very ambitious in its aims and developed a model, perhaps too fast and too dominated by one major institution, NIMHANS. Ownership of the programme at central, state and district levels suffered as a consequence. In the early years (late 1970s-early 1980s) very few mental health initiatives existed - the Bellary model was the best available at the time. However, several generations of psychiatrists since then have retained the same vision of the DMHP, and have romanticised the initial model and insufficiently questioned it. This possibly led to less creativity or inspiration from other models (such as NGO models) to adapt the DMHP programme. WHO have summarised the evidence to suggest using a collaborative and integrated model of delivering mental health care through primary care, but the degree to which the DMHP followed this has been doubtful. All the elements the WHO recommended to ensure successful integration have not occurred (adequate specialist and primary care staff, regular supplies of essential psychotropic drugs, linkages with specialist care services, referral criteria) or even been considered (developing information and communications systems, appropriate links with other community and social services) – these are also common failings in many LMICs [59,60].

In addition, integration requires more than education of providers or the addition of services. It demands a “new perspective which engages an orientation towards the unique mental, physical, social and cultural needs of the individual”, and involves family and community support [61]. India’s NMHP has prioritised mental health literacy of the general population through campaigns but has not re-orientated the primary care provider, either the doctor or the lay health worker, away from a biomedical model to a process of thinking necessary for comprehensive mental and physical care. India may consider remedying this, as have some of its low- and middle- income counterparts, where this re-orientation of health workers is being attempted for example in South Africa (with primary care nurses and health district management) and in Mozambique (with traditional healers) [61-63].

This study highlighted that the implementation of the model has been poor at several levels, particularly at the human resource level. As a middle-income country, and being the 5th largest economy in the World, India should have sufficient resources to provide sufficient mental health specialists and primary health specialists for at least the basic provision of consultation liaison with primary care [64]. However, not only are too few specialist and non-specialist workforce trained, but they are poorly distributed and favour working in the private sector or moving abroad.

One glaring omission in the discussion about increasing human resources within the DMHP- both in the literature and amongst participants interviewed - is the lack of thought and initiative as to how to incorporate the large private mental health sector in India to overcome the lack of specialists, particularly as public health services in India only cover 20-30% of the population [23]. There is growing concern in both high-income countries (like the USA model) and LMICs (such as the Chilean mental health reforms) whether partnering with the private sector contributes to inequity of care [61,65]. However given the dearth of manpower in India, the option should be considered. Within the health sector, the National Rural Health Mission and other sectors (TB control programmes, surgical procedures, hospital ventures) have encouraged public private partnership development with successful examples mainly with the not-for-profit private sector (e.g. NGOs). Psychiatry being relatively less technology-intensive has had less private involvement as the business models are less robust, are too regulated or are stigmatised. Several caveats also exist to incorporating the for-profit or not-for-profit private sectors. Due to the federal system in India, the decision to accept or promote such partnerships is devolved to each individual State. Other caveats include the private sector’s motives, incentivisation, and ensuring adequate governance and monitoring arrangements [66].

In addition, primary care workers have received overall ineffective training and insufficient supervision, and no solution has been implemented to get specialists onboard or to ensure a sturdy state- and national- leadership. These weaknesses have been reinforced by poor mechanisms to evaluate the programme and to ensure accountability, which have meant there is no certainty of the quality of care provided or of patient outcomes. These problems are common to many LMICs [67]. For example in South Africa, despite a decentralisation model which promotes integration of mental health into primary health care, there is a paucity of community-based mental health resources and the same problems of poor identification and treatment of mental disorders by primary care physicians. It also has problems of support, supervision and of providing more than just an emergency reactive service [68].

However, the above criticism of the NMHP/DMHP’s implementation was the result of contextual barriers. The main problem over the years has been to convince policy makers about the public health importance of mental health. Despite the success of some early leaders in lobbying for increased funding for mental health care, the second main hurdle has been the system-wide barriers, the bureaucratic and political hurdles, drug supply issues and the need to strengthen health systems. These required interventions outside the NMHP and were difficult to address by the small group of specialists spearheading the programme. These drawbacks are the case for the whole health sector in India but, programmes which have been successful in overcoming such barriers are those which have had more political and financial support and more structured leadership (such as HIV care and maternal and child health care). Integration of programmes is feasible, and therefore should be achievable for mental health care if the appropriate financing and implementation ingredients are present. For example given the rising burden of non-communicable diseases, more resources could be leveraged for integrated mental health care from the chronic care service delivery platform which is growing in India as it is elsewhere.

### How does this history shed light on current policy recommendations?

In the last three years, a group of experts has been commissioned to advise the government on priorities for the next funding cycle, the 12th Five Year Plan. This reflects a growing political commitment to mental health. The experts conducted intensive investigations into NMHP implementation across India [69]. Their main recommendations feature in Table 3.

**Table 3:** Mental Health Policy Group key recommendations

**Area of recommendation**	Summary of recommendations*
Programme management	Ensure a clear structure for funding, management and coordination of teams at central, state and district levels. Promote intra- and inter-sectoral collaborations.
Community involvement	Improve accountability and local ownership of the DMHP. Promote more participation of NGO/private sector.
Technical support	Provide an overarching technical support and advisory group (TSAG) for all the States which will provide mentoring to districts to help with implementation difficulties.
Revitalising human resources:	Provide technical and quality inputs to increase the number of specialist resources (through relaxing educational requirements). Introduce a new cadre, a community mental health worker to identify, treat, provide basic counselling, and help access social benefits. Improve training.
Ensure quality of care is provided	Improve systems for monitoring, evaluation, operational research, a mental health information system, adequate supply of medicines, continuity of care in the community, user/carer involvement in decision making.
Incorporate life skills education and improve current preventative and promotive services	Create collaborations with other concerned departments (such as education).
Extend services to urban areas	Include the provision of a community mental health worker.

*Based on recommendations provided in reference [69].

Two authors of this paper (VP and SJ) were part of this policy group, but we discuss here to what extent the views of our interviewees correlated with these recommendations. Participants broadly agreed with the recommendations but their experiences over the last 30 years put a different emphasis on priority areas. They highlighted continuity of leadership. The lack of continuity in government officials, not just their lack of technical and managerial skills, meant the same lessons were constantly relearned. We suggest here that the challenge to improving continuity would need to start with sensitising, attracting and retaining specialists to be leaders, managers and supervisors. Our analysis also highlighted common barriers of political and bureaucratic hurdles. Politics and hierarchical power structures could be minimised with safeguards at policy level but also a more democratic and locally accountable system (such as through the Panchayati Raj as is done in the Southern and North-Eastern States).

PHC doctors are currently overburdened (as are many other government primary care employees). Interviewees did not agree whether a new cadre of community worker might be required to deliver mental health care. The nature of this new cadre is also debatable. The mental health policy group’s suggestion to add two community mental health workers to each existing PHC team seems to be potentially unrealistic given human resource shortages. The post of chronic disease worker (a social worker or a lay health worker) who coordinated, counselled and provided psychosocial support for all chronic diseases, might be more sustainable in light of the growing non-communicable disease burden and would be better integrated in primary care, rather than setting up an exceptional service for mental health care [70].

Interviewees identified the importance of the quality of health providers (PHC workers and specialists), their motivation and competence. Suggestions for improving PHC workers’ competence included changing training to being skills- and problem-based and having more supervision, ongoing training and monitoring. This would be subject to sufficient mental health professionals joining the DMHP. This major specialist manpower caveat may be resolved by better incentives increasing their confidence in the programme and belief in integrated care, and improvements in supervision. These ideas have also been voiced by government reports [34] but they are still to be implemented and will require strong leadership to make them happen.

## Conclusion

At this important juncture in time, as the 12th Five Year Plan is in preparation, the history of the last 30 years cautions policymakers about the visible poor investment in programme implementation and innovation, which has led to stagnation and reinventing the wheel. The reasons for not achieving adequate implementation are not necessarily failures that could have been entirely avoided. The mindset at the time (such as professional conflicts), and external hurdles influencing the NMHP (such as political neglect, funding problems, patronage) were important barriers which could not be controlled by NMHP advocates or leaders. These factors cannot be changed by adjusting the model as much as by encouraging important stakeholders (central and state governments) for acceptance, financing and technical support for the elements that would make the integration of the DMHP into primary care successful. Amongst the most important elements, programme leadership needs rethinking to have better continuity and to ensure better management at district, state and national levels. This would necessitate more commitment and collaboration between the ministry of health, primary health programmes and mental health professionals.

Given the growing interest in primary mental health care within India and globally, lessons learned from prior policy and programme challenges, which are often similar to those in other LMICs, should play a stronger role in informing current policy.

## Abbreviations

DMHP: District Mental Health Programme; GP: General practitioner; HIV: Human immunodeficiency virus; LMICs: Low- and middle-income countries; NGO: Non-governmental organisation; NIMHANS: National institute for mental health and neurosciences; NMHP: National Mental Health Programme; NRHM: National Rural Health Mission; PHC: Primary health centre; PHWs: Primary health workers; WHO: World Health Organisation.

## Competing interests

VP and SJ are members of the Mental Health Policy Group which has provided recommendations to the Central Government of India recommendations on restructuring the DMHP for the 12th Five Year Plan.

## Authors’ contributions

NvG conceived of the study, participated in its design and coordination, carried out the interviews and drafted the manuscript. VB guided NvG in the conception and design of the study, and supervised her work. VP also helped with conception of the study and helped choose relevant participants for the study. VB, SJ and VP provided guidance on analysis and interpretation of the material. All authors read and approved the final manuscript.
